# Evaluating the Effectiveness of Social Distancing Interventions to Delay or Flatten the Epidemic Curve of Coronavirus Disease

**DOI:** 10.3201/eid2608.201093

**Published:** 2020-08

**Authors:** Laura Matrajt, Tiffany Leung

**Affiliations:** Fred Hutchinson Cancer Research Center, Seattle, Washington, USA

**Keywords:** respiratory infections, severe acute respiratory syndrome coronavirus 2, SARS-CoV-2, SARS, COVID-19, 2019 novel coronavirus disease, coronavirus disease, zoonoses, viruses, coronavirus, disease transmission, mathematical model, social distancing interventions, nonpharmaceutical interventions

## Abstract

By April 2, 2020, >1 million persons worldwide were infected with severe acute respiratory syndrome coronavirus 2. We used a mathematical model to investigate the effectiveness of social distancing interventions in a mid-sized city. Interventions reduced contacts of adults >60 years of age, adults 20–59 years of age, and children <19 years of age for 6 weeks. Our results suggest interventions started earlier in the epidemic delay the epidemic curve and interventions started later flatten the epidemic curve. We noted that, while social distancing interventions were in place, most new cases, hospitalizations, and deaths were averted, even with modest reductions in contact among adults. However, when interventions ended, the epidemic rebounded. Our models suggest that social distancing can provide crucial time to increase healthcare capacity but must occur in conjunction with testing and contact tracing of all suspected cases to mitigate virus transmission.

Severe acute respiratory syndrome coronavirus 2 (SARS-CoV-2) emerged in Wuhan, China, in December 2019 (*1*), and in March 2020, the World Health Organization declared coronavirus disease (COVID-19) a pandemic (*2*). By April 2, 2020, COVID-19 had spread to >181 countries worldwide, and >1 million confirmed cases of COVID-19 and >50,000 deaths had been reported globally (*3*).

On January 21, 2020, the first case of COVID-19 in the United States was identified in a traveler who had recently returned to Washington from Wuhan (*4*,*5*). By March 14, Washington had reported 642 confirmed cases and 40 deaths associated with COVID-19 (*6*). In response to the rapid spread of the virus, on March 12, 2020, approximately 7 weeks after the first confirmed case in the state, the governor of Washington announced a set of interventions in 3 counties (7,8). More stringent prohibitions were soon imposed, followed by a shelter-in-place order lasting >6 weeks beginning on March 25, 2020 (*9*). Similar interventions have been enacted in other US states and in countries in Europe (*10*,*11*,*12*).

We used an epidemic mathematical model to quantify the effectiveness of social distancing interventions in a medium-sized city in the United States or Europe by using Seattle, Washington, as an example. We provide estimates for the proportion of cases, hospitalizations, and deaths averted in the short term and identify key challenges in evaluating the effectiveness of these interventions.

## Methods

**Figure 1:**
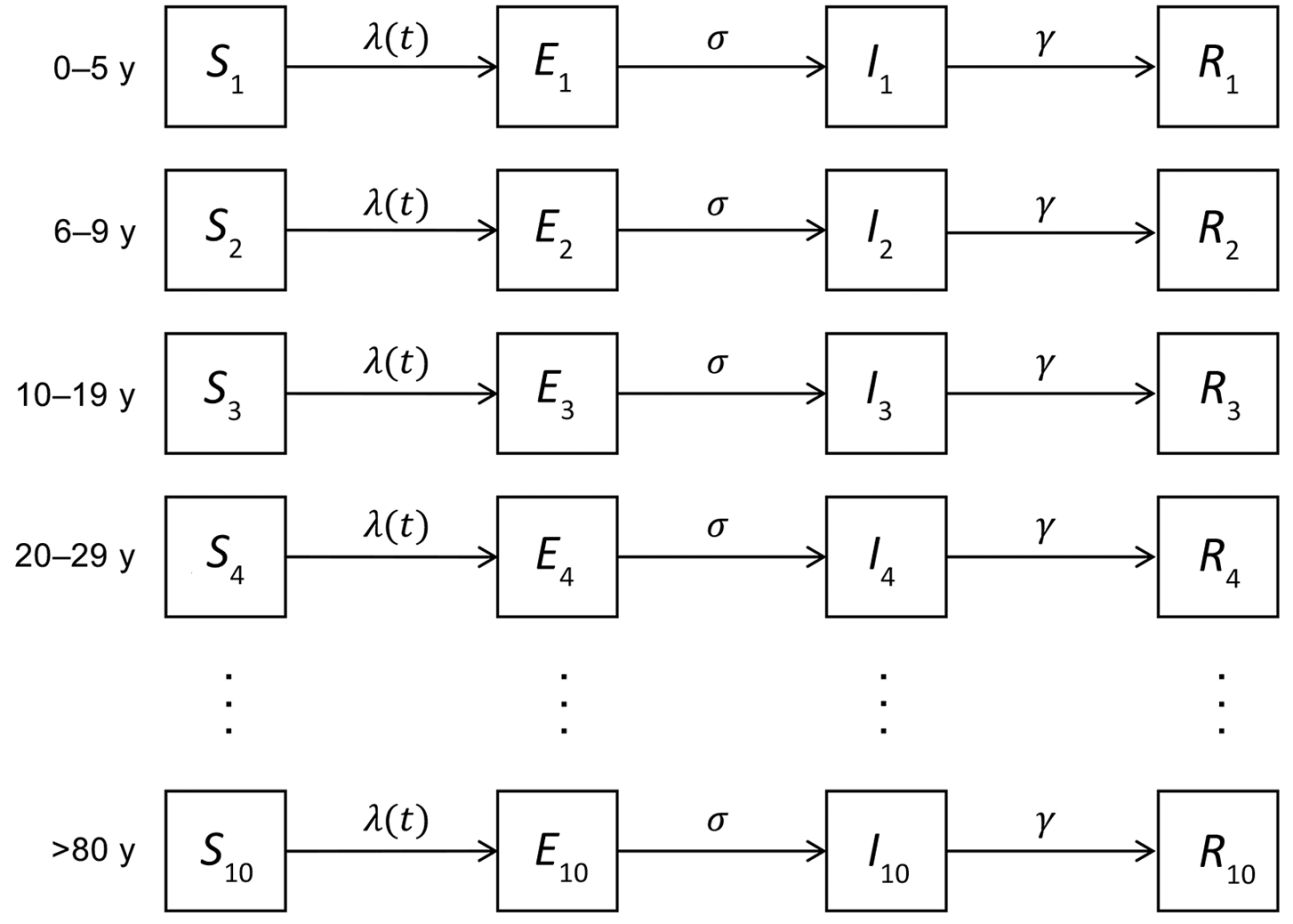
Mathematical model illustrating study population divided into 10 age groups and stratified as susceptible (*S*), exposed (*E*), infectious (*I*), and removed (*R*) from coronavirus disease epidemic. Susceptible persons become exposed at the force of infection *λ*(*t*), progress to become infectious at rate, *σ*, and are removed from infecting others at rate, *γ*.

We developed an age-structured susceptible-exposed-infectious-removed model to describe the transmission of SARS-CoV-2 (Appendix). We divided the population into 10 age groups: 0–5, 6–9, 10–19, 20–29, 30–39, 40–49, 50–59, 60–69, 70–79, and >80 years of age. We calibrated the model to the age distribution of the population of the Seattle metropolitan area by using data from the US Census Bureau (*13*). For each age group, we divided the population into compartments: susceptible (*S*) for persons who could be infected; exposed (*E*) for persons who have been exposed but are not yet infectious; infectious (*I*); and removed (*R*) for persons who have recovered or died (Table; Figure 1). We only considered symptomatic infections on the basis of estimates that <1% of infections are asymptomatic (*15*). We assumed only 20% of the cases would be identified because 80% of cases are reported to be mild and would probably be undocumented (*16*,*17*). We used previously reported case-fatality and hospitalization rates by age group (*16*,*18*). We used the contact matrix for 6 age groups computed by Wallinga et al. (*19*) and extended it to 10 age groups (Appendix). 

**Table:** Description of parameters used in the susceptible-exposed-infectious-removed mathematical model for evaluating the effectiveness of social distancing interventions on coronavirus disease*

Parameter	Meaning	Value	Range	Reference
1/σ	Mean latent period	5.16 days	4.5–5.8 days	(*14*)
1/γ	Mean infectious period	5.02 days	3–9 days	†
β	Transmission coefficient	Calculated	NA	NA
С	Contact matrix	NA	NA	(*19*)
N	Total population	3.5 million	NA	(*13*)
*NA, not applicable. †Q. Bi, unpub. data, https://www.medrxiv.org/content/10.1101/2020.03.03.20028423v3.				

We used January 21, 2020, the day the first case was identified in Washington, as the first day of our simulation on the basis of the analysis by T. Bedford (*20*). By using genomic epidemiology of the first 2 COVID-19 cases identified in Washington, Bedford found that SARS-CoV-2 had been circulating locally for 6 weeks before the second case was identified in the state (*20*). 

We modeled social distancing by reducing the contact rates in an age group for 6 weeks, corresponding to the policy in Washington in mid-March (*7*,*8*,*21*). We divided the population into 3 major groups for social distancing interventions: children, persons <19 years of age; adults 20–59 years of age; and adults >60 years of age.

We investigated the effectiveness of 4 scenarios of social distancing. The first was distancing only for adults >60 years of age, in which contacts for this group were reduced by 95%. The rationale for this scenario is that older adults are at highest risk for hospitalization and death and should have the most drastic restrictions in their contacts. Similar policies were implemented in early April in some countries, such as Sweden (*22*). In the second scenario, adults >60 years of age would reduce social contacts by 95% and children would reduce contacts by 85%, assuming that most of the contacts of children occur at school and would be reduced due to school closures. This scenario corresponds to an intervention in which the high-risk group is fully protected. In addition, it reduces the contact rates for children, who are known to be a major part of the chain of transmission for other respiratory infectious diseases. Research indicates that children are infected with SARS-CoV-2 as often as adults (Q. Bi, unpub. data, https://www.medrxiv.org/content/10.1101/2020.03.03.20028423v3) but seem to have much milder symptoms (*23*). At this point, whether their infectiousness also is reduced is unclear. In the third scenario, adults >60 years of age reduce contacts by 95% and adults <60 years of age reduce contacts by 25%, 75%, or 95%. This scenario corresponds to a policy in which high-risk age groups still are protected and younger adults are somewhat restricted in their contacts. However, persons in essential businesses can continue working and children can resume school, which is crucial considering school closures have been shown to have an adverse effect on the economy (*24*). In the fourth scenario, contacts are reduced for every group; adults >60 years of age reduce contacts by 95%, children by 85%, and adults <60 years of age by 25%, 75%, or 95%. This scenario represents many interventions currently in place throughout the world.

To quantify the uncertainty around our results, we performed 1,000 simulations varying 3 parameters: the basic reproduction number (R_0_), the latent period, and the duration of infectiousness (Appendix). For each statistic in the results, we computed the error bars by removing the top and bottom 2.5% of the simulations.

## Results

Estimates for the duration of infectiousness for SARS-CoV-2 range from 5 to 20 days (*25*; Q. Bi, unpub. data, https://www.medrxiv.org/content/10.1101/2020.03.03.20028423v3). Therefore, we analyzed the influence of the duration of infectiousness on the effectiveness of the social distancing interventions. We kept all other parameters fixed but considered an epidemic with infectious periods of 5, 6, 7, or 8 days, which correspond to the most plausible values (*25*; Q. Bi, unpub. data, https://www.medrxiv.org/content/10.1101/2020.03.03.20028423v3). 

**Figure 2:**
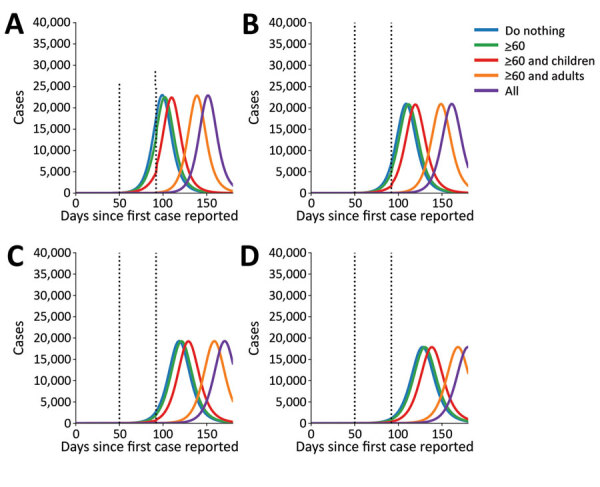
Number of ascertained coronavirus disease cases over 180 days (identified cases over time calculated by mathematical model) using varying infectious periods: A) 5 days; B) 6 days; C) 7 days; D) 8 days. We used parameter values of R_0_ = 3, *γ* = 1/5.02, *σ* = 1/5.16, and contact in adults reduced by 75%. Dotted lines indicate the beginning of the social distancing intervention at 50 days and end at 92 days.

In our model, when the infectious period was set to a shorter time of 5 days, the epidemic peaked at 100 days after the introduction of the first case. As we extended the infectious period, the epidemic took much longer to take off (Figure 2) because we kept a fixed R_0_, so that a longer infectious period implied a smaller infectious rate. When we used the longest infectious period of 8 days, we noted the epidemic peaked 128 days after the first case was introduced. Therefore, early interventions delay the epidemic but do not substantially change the pool of susceptible persons, which allows similar-sized epidemics to occur later (Figure 2).

We then considered the delay of the epidemic under the 4 social distancing interventions and different infectious periods (Figure 2). As expected, the fourth social distancing strategy, the one applied to all age groups, delayed the epidemic the longest, >50 days, compared with a baseline of using no interventions. Targeting adults >60 years of age and children delayed the epidemic by ≈10 days, regardless of infectious period. Targeting adults <60 and >60 years of age delayed the epidemic by 41 days when we set the infectious period to 8 days and delayed it 39 days when we set the infectious period to 5 days. Social distancing of only adults >60 years of age only delayed the epidemic by 2 days, regardless of infectious period (Appendix Table 1). The infectious period did not substantially affect the peak epidemic height compared with baseline.

**Figure 3:**
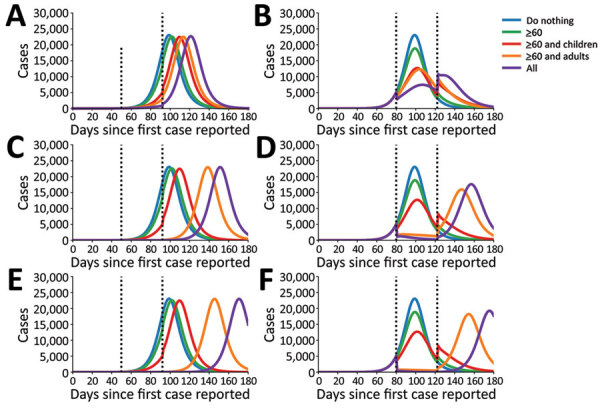
Number of ascertained coronavirus disease (identified cases over time calculated by mathematical model) with adults reducing their contact by 25% (A, B); 75% (C, D); and 95% (E, F). We used parameter values of R_0_ = 3, *γ* = 1/5.02, *σ* = 1/5.16. Dotted lines represent the beginning and end of the 6-week social distancing interventions, after which contact rates return to normal. For panels A, C, and E, intervention starts at day 50 after identification of first case; for panels B, D, and F, intervention starts at day 80 after identification of first case.

We examined the effectiveness of the 3 social distancing interventions in adults and the timeframe in which interventions began. We considered social distancing interventions starting 50 days (Figure 3, panels A, C, E) and 80 days (Figure 3, panels B, D, F) after the first case was identified and reduction in adult contacts by 25% (Figure 3, panels A, B), 75% (Figure 3, panels C, D), and 95% (Figure 3, panels E, F). We found that the effect of interventions was dramatically different when started early in the epidemic curve, before the exponential phase of the epidemic, rather than later.

When we started interventions on day 50, we saw a delay in the epidemic regardless of the level of reductions in contact in the adult population, with little change in the magnitude of the epidemic peak. In comparison, when we began the interventions later, during the exponential phase of the epidemic, all interventions flattened epidemic curve. The strategy of reducing the contacts only of adults >60 years of age resulted in a moderate reduction of 5,000 (21%) fewer cases at the epidemic peak compared with baseline. Limiting contact for adults >60 years of age, as expected, is the only intervention for which there was minimal rebound after the intervention was lifted (Figure 3, panels B, D, F) because older adults make up only 16% of the population and have substantially fewer contacts than the other age groups.

We found that the strategy targeting adults >60 years of age and children resulted in 10,500 (45%) fewer cases than baseline at the epidemic peak (Figure 3, panels B, D, F), emphasizing the fact that children are the age group with the highest number of contacts in our model. By comparison, when we applied the adults-only strategy, we saw 11,000 (47%) fewer cases than baseline at the epidemic peak for 25% reduction in contacts in adults <60 years of age (Figure 3, panel B). When we reduced contact by 75% in this age group, the peak epidemic cases dropped by 21,000 (91%). When we reduced contact by 95% in this age group, we noted 22,500 (98%) fewer cases (Figure 3, panels D, F), and the epidemic curve grew at a slower rate in both instances. Of the 4 intervention scenarios, the strategy involving all age-groups decreased the epidemic peak the most and showed the slowest growth rate, which we expected because contacts in all age groups are reduced. Even when we used a lower reduction in contacts of 25% in adults <60 years of age, we noted 16,000 (69%) fewer cases at the epidemic peak (Figure 3, panel B). With higher reduction in contacts (95%) in adults <60 years of age, the strategy involving all age groups mitigated nearly all cases at the epidemic peak (Figure 3, panel F). However, our results suggest that all interventions can result in new epidemic curves once the interventions are lifted.

**Figure 4:**
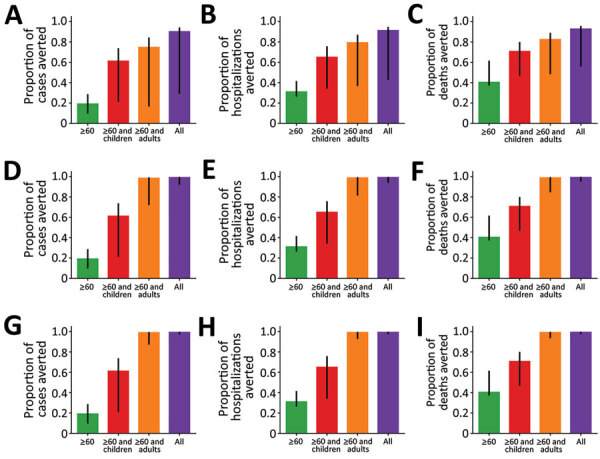
Proportion of coronavirus disease cases, hospitalizations, and deaths averted during 100 days for various social distancing scenarios in which adults reduce their contact by 25% (A–C); 75% (D–F); and 95% (G–I). We used parameter values of R_0_ = 3, *γ* = 1/5.02, *σ* = 1/5.16. Error bars represent results of 1,000 parameter simulations with the top and bottom 2.5% simulations removed.

Next, we considered the effects of social distancing interventions over the first 100 days of the epidemic and assumed that the social distancing interventions started on day 50, which corresponds to the approximate date when social distancing interventions started in Washington. To investigate the sensitivity of the model to the chosen parameters, we ran 1,000 simulations (Appendix). We obtained curves that varied widely for both the number of cases and the duration and timing of the epidemic (Appendix Figures 1–3). We ran simulations with the mean parameter values (R_0_ = 3, an infectious period lasting 5 days, and a latent period of 5.1 days). We then observed the number of cases and proportion of cases, hospitalizations, and deaths averted during the first 100 days. We noted that reducing the contacts of adults >60 years of age averted only 18% of cases for the whole population (Figure 4) but averted 50% of cases for this age group (Appendix Figure 4). In addition, this intervention reduced the overall number of hospitalizations by 30% and reduced deaths by 39% for the whole population (Figure 4) and hospitalizations and deaths by >49% for the adults >60 years of age (Appendix Figures 5, 6). Adding a social distancing intervention in children slowed the epidemic curve (Figure 3) and reduced the overall hospitalizations by >64% (Figure 4) and by >53% across all age groups (Appendix Figures 5, 6).

When only 25% of adults <60 years of age changed their contact habits, all interventions rebounded as soon as the intervention was lifted (Figure 3, panel A). Surprisingly, cases, and hence hospitalizations and deaths, can be reduced by 90% during the first 100 days if all groups reduce their contacts with others, even when adults do so by only 25% (Figure 4, panel A). In this scenario, the reduction in the number of cases, hospitalizations, and deaths was evenly distributed across all age groups (Appendix Figure 4, panel A, Figure 5, panel A, Figure 6, panel A). When adults <60 years of age reduced contacts by 75%, cases, hospitalizations, and deaths rebounded immediately after the end of the intervention, except in the intervention in which contact was reduced for all groups (Figure 3, panel C). As expected, adult groups had the greatest reductions in cases, hospitalizations, and deaths from this intervention (Appendix Figure 5, panel B, Figure 6, panel B). When adults <60 years of age reduced contacts by >75%, the strategies that reduced the contacts of adults only and that reduced the contacts of everyone averted >98% of cases, hospitalizations and deaths during the first 100 days (Figure 4, panels E, F). Further, when we reduced the contact rate of adults by >75%, the strategy targeting all adults and the strategy targeting everyone mitigated the outbreak (Figure 3, panels C, E; Figure 4; Appendix Figure 4, panels B, C, Figure 5, panels B, C, Figure 6, panels B, C). However, our model suggests that the epidemic would rebound even in these scenarios. Of note, the error bars were much larger when adults reduced their contact rates by 25%, and this uncertainty tended to smooth out as the adults further reduced their contact rates.

## Discussion

The term “flatten the curve,” originating from the Centers for Disease Control and Prevention (*26*), has been used widely to describe the effects of social distancing interventions. Our results highlight how the timing of social distancing interventions can affect the epidemic curve. In our model, interventions put in place and lifted early in the epidemic only delayed the epidemic and did not flatten the epidemic curve. When an intervention was put in place later, we noted a flattening of the epidemic curve. Our results showed that the effectiveness of the intervention will depend on the ratio of susceptible, infected, and recovered persons in the population at the beginning of the intervention. Therefore, an accurate estimate of the number of current and recovered cases is crucial for evaluating possible interventions. As of April 2, 2020, the United States had performed 3,825 tests for SARS-CoV-2 per 1 million population (*27*). By comparison, Italy had performed 9,829 tests/1 million population (*27*). Expanding testing capabilities in all affected countries is critical to slowing and controlling the pandemic.

Some evidence suggests that persons who recover from COVID-19 will develop immunity to SARS-CoV-2 (*28*). However, at this point the duration of immunity is unclear. If immunity lasts longer than the outbreak, then waning immunity will not affect the dynamics of the epidemic. Furthermore, persons who recover from COVID-19 could re-enter the workforce and help care for the most vulnerable groups. However, if immunity is short-lived, for instance on the order of weeks, persons who recover could become re-infected, and extensions to social distancing interventions might be necessary.

We used a mathematical model to quantify short-term effectiveness of social distancing interventions by measuring the number of cases, hospitalizations, and deaths averted during the first 100 days under 4 social distancing intervention scenarios and assuming different levels of reduction in the contacts of the adult population. When we investigated the short-term effects of social distancing interventions started early in the epidemic, our models suggest that the intervention involving all age groups would consistently decrease the number of cases considerably and delay the epidemic the most. Of note, with >25% reduction in contact rates for the adult population, combined with 95% reduction in older adults, the number of hospitalizations and deaths could be reduced by >78% during the first 100 days, a finding that agrees with previous reports (*29*,*30*). 

Our results must be interpreted with caution. Hospitalizations and deaths averted during the first 100 days in our model would likely occur later if the interventions are lifted without taking any further action, such as widespread testing, self-isolation of infected persons, and contact tracing. As in any model, our assumptions could overestimate the effect of the interventions. However, quantifying the short-term effects of an intervention is vital to help decision makers estimate the immediate number of resources needed and plan for future interventions. 

Our simulations suggest that even in the more optimistic scenario in which all age groups reduce their contact rates by >85%, the epidemic is set to rebound once the social distancing interventions are lifted. Our results suggest that social distancing interventions can give communities vital time to strengthen healthcare systems and restock medical supplies, but these interventions, if lifted too quickly, will fail to mitigate the current pandemic. Other modeling results also have suggested that extended periods of social distancing would be needed to control transmission (*18*). However, sustaining social distancing interventions over several months might not be feasible economically and socially. Therefore, a combination of social distancing interventions, testing, isolation, and contact tracing of new cases is needed to suppress transmission of SARS-CoV-2 (*31*,*32*). In addition, these interventions must happen in synchrony around the world because a new imported case could spark a new outbreak in any given region.

Our results suggest that the SARS-CoV-2 infectious period has an extraordinary influence in the modeled speed of an epidemic and in the effectiveness of the interventions considered. However, current estimates of the infectious period range from 5 to 20 days (*25*; Q. Bi, unpub. data, https://www.medrxiv.org/content/10.1101/2020.03.03.20028423v3). Of note, all estimates of the infectious period were made on the basis of PCR positivity, which does not necessarily translate to viability or infectivity of the virus (*33*). We urgently need studies to definitively define the duration of infectiousness of SARS-CoV-2.

Our work has several limitations and should be interpreted accordingly. First, deterministic mathematical models tend to overestimate the final size of an epidemic. Further, deterministic models always will predict a rebound in the epidemic once the intervention is lifted if the number of exposed or infectious persons is >0. To avoid that problem, we forced our infected compartments to 0 if they had <1 person infected at any given time. Second, we considered the latent period to be equal to the incubation period, but others have suggested that presymptomatic transmission is occurring (L. Tindale, unpub. data, https://www.medrxiv.org/content/10.1101/2020.03.03.20029983v1) and SARS-CoV-2 is shed for a prolonged time after symptoms end (*34*). Whether virus shed by convalescent persons can infect others currently is unclear. Further, we considered that mild and severe cases would be equally infectious and our model could be overestimating the total number of infections, which would amplify the effect of social distancing interventions. We also considered infected children and adults to be equally infectious, and our model could be overestimating the effect of social distancing in persons <19 years of age. Strong evidence suggests that children have milder COVID-19 symptoms than adults and might be less infectious (*23*). More studies are needed clarify the role children play in SARS-CoV-2 transmission. In our models, we assumed death and hospitalization rates would be similar to those experienced in Wuhan, where older age groups have been the most affected. Because different countries have different population structures and different healthcare infrastructure, including varying numbers of hospital beds, ventilators, and intensive care unit beds, effects of social distancing interventions could vary widely in different places. 

Our results align with an increasing number of publications estimating the effects of interventions against COVID-19. Several researchers have investigated how social distancing interventions in Wuhan might have affected the trajectory of the outbreak (*30*,*35*,*36*; J. Zhang, unpub. data, https://www.medrxiv.org/content/10.1101/2020.03.19.20039107v1). Others have investigated the effect of similar measures elsewhere and concluded that social distancing interventions alone will not be able to control the pandemic (*37**,**38*; M.A. Acuña-Zegarra, unpub. data, https://www.medrxiv.org/content/10.1101/2020.03.28.20046276v1; N.G. Davies, unpub. data, medrxiv.org/content/10.1101/2020.04.01.20049908v1; S. Kissler, unpub. data, https://www.medrxiv.org/content/10.1101/2020.03.22.20041079v1). 

Taken together, our results suggest that more aggressive approaches should be taken to mitigate the transmission of SARS-CoV-2. Social distancing interventions need to occur in tandem with testing and contact tracing to minimize the burden of COVID-19. New information about the epidemiologic characteristics of SARS-CoV-2 continues to arise. Incorporating such information into mathematical models such as ours is key to providing public health officials with the best tools to make decisions in uncertain times.

Appendix Additional information on evaluating effectiveness of social distancing interventions against coronavirus disease.
