# Supplementary material for: Evaluating the Effectiveness of Social Distancing Interventions to Delay or Flatten the Epidemic Curve of Coronavirus Disease
Source: Emerg Infect Dis. 2020 Aug;26(8):1740–8. doi: 10.3201/eid2608.201093 (PMC7392458; doi:10.3201/eid2608.201093)
Supplement: Appendix — Additional information on evaluating effectiveness of social distancing interventions against coronavirus disease. [file 20-1093-Techapp-s1.pdf]

Article title: https://doi.org/10.3201/eid2608.201093

Evaluating the Effectiveness of Social Distancing Interventions against Coronavirus Disease

Appendix

The Model

We considered an age-structured susceptible-exposed-infectious-removed (SEIR) model with 10 discrete age groups: 0–5, 6–9, 10–19, 20–29, 30–39, 40–49, 50–59, 60–69, 70–79, and ≥80 years old. The equations for the model are as follows:

$$\frac{dS_i}{dt} = -\lambda S_i ,$$
$$\frac{dE_i}{dt} = \lambda S_i - \sigma E_i ,$$
$$\frac{dI_i}{dt} = \sigma E_i - \gamma I_i ,$$
$$\frac{dR_i}{dt} = \gamma I_i ,$$

where the state variables S_i, E_i, I_i, and R_i represent the numbers of susceptible, exposed, infectious, and removed persons in each age group i. Removed persons are those who can no longer infect others. The force of infection is represented by $\lambda = \sum_{j=1}^{10} \beta \frac{c_{ij}}{N} I_j$, where c_{ij} corresponds to the estimated number of contacts per day between age class i and j in a total population size N. Based on current estimates that put <1% of the infections as asymptomatic (*1*), we considered only symptomatic infections. Details of parameters values can be found in the Table of the main text.

To quantify the uncertainty around our results, we performed 1,000 simulations varying 3 parameters: the basic reproduction number, R_0; the latent period; and the duration of infectiousness. By using current estimates (*2*,*3*,*4*; L. Tindale, unpub. data, https://www.medrxiv.org/content/10.1101/2020.03.03.20029983v1), we sampled values of R_0

from a truncated normal distribution with mean 3 ranging from 2–4. We assumed that the latent period was similar to the incubation period and assumed a gamma distribution with mean 5.1 days and SD 0.7 (*5*). We sampled the duration of infectiousness from a truncated normal distribution with mean 5 days and SD 0.7 ranging from 3–9 (*6*; Q. Bi, unpub. data, https://www.medrxiv.org/content/10.1101/2020.03.03.20028423v3). For each R_0, infectious period, and latent period considered, we used the next-generation matrix approach (*7*) to calculate the transmission coefficient β.

References

1. Wu Z, McGoogan JM. Characteristics of and important lessons from the coronavirus disease (COVID-19) outbreak in China: summary of a report of 72,314 cases from the Chinese Center for Disease Control and Prevention. JAMA. 2020;323:1239–42. PubMed https://doi.org/10.1001/jama.2020.2648

2. Li Q, Guan X, Wu P, Wang X, Zhou L, Tong Y, et al. Early transmission dynamics in Wuhan, China, of novel coronavirus-infected pneumonia. N Engl J Med. 2020;382:1199–1207. PubMed https://doi.org/10.1056/NEJMoa2001316

3. Wu JT, Leung K, Leung GM. Nowcasting and forecasting the potential domestic and international spread of the 2019-nCoV outbreak originating in Wuhan, China: a modelling study. Lancet. 2020;395:689–97. PubMed https://doi.org/10.1016/S0140-6736(20)30260-9

4. Zhao S, Lin Q, Ran J, Musa SS, Yang G, Wang W, et al. Preliminary estimation of the basic reproduction number of novel coronavirus (2019-nCoV) in China, from 2019 to 2020: A data-driven analysis in the early phase of the outbreak. Int J Infect Dis. 2020;92:214–7. 10.1016/j.ijid.2020.01.050 PubMed https://doi.org/10.1016/j.ijid.2020.01.050

5. Lauer SA, Grantz KH, Bi Q, Jones FK, Zheng Q, Meredith HR, et al. The incubation period of coronavirus disease 2019 (COVID-19) from publicly reported confirmed cases: estimation and application. Ann Intern Med. 2020 Mar 10 [Epub ahead of print]. PubMed https://doi.org/10.7326/M20-0504

6. Zou L, Ruan F, Huang M, Liang L, Huang H, Hong Z, et al. SARS-CoV-2 viral load in upper respiratory specimens of infected patients. N Engl J Med. 2020;382:1177–9. PubMed https://doi.org/10.1056/NEJMc2001737

7. van den Driessche P, Watmough J. Reproduction numbers and sub-threshold endemic equilibria for compartmental models of disease transmission. Math Biosci. 2002;180:29–48. PubMed https://doi.org/10.1016/S0025-5564(02)00108-6

Appendix Table. Delay in coronavirus disease (COVID-19) epidemic peaks under different social distancing measures for varying infectious periods and age groups*

	Delay in epidemic peak, d (vs. no intervention, d)			
	Infectious period, d			
Persons limiting contact	5	6	7	8
None	100	110	118	128
≥60 y only	102 (2)	112 (2)	120 (2)	130 (2)
≥60 y and children	110 (10)	120 (10)	129 (11)	139 (11)
Adults only	139 (39)	149 (39)	159 (41)	169 (41)
All	152 (52)	161 (51)	171 (53)	>180 (>52)

*This table corresponds to Figure 2 in the main text. Age groups are as follows: children, persons ≤19 years of age; adults, persons 20–59 years of age; and persons ≥60 years of age.

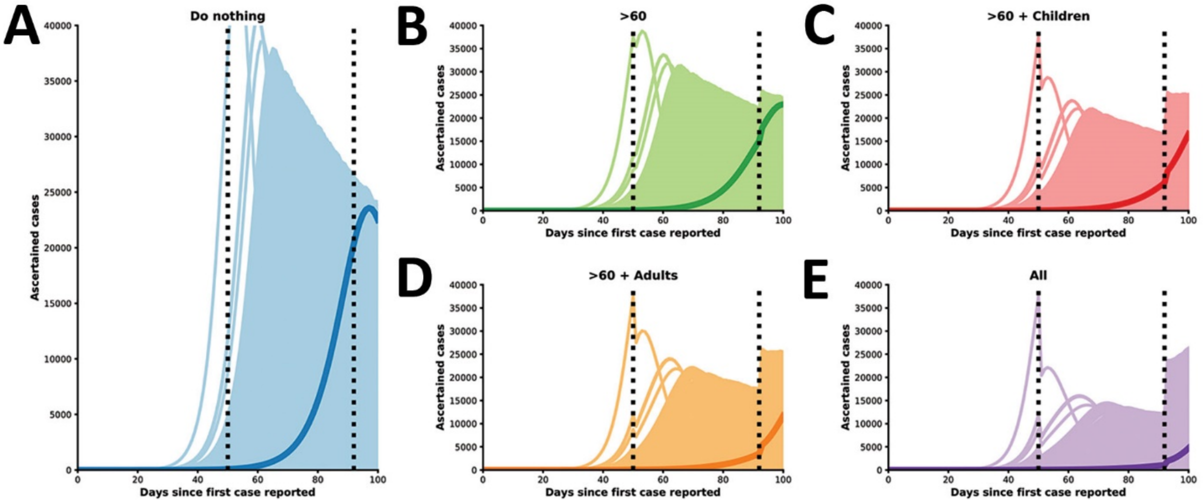

Appendix Figure 1. Number of ascertained coronavirus disease (COVID-19) cases for 5 scenarios of social distancing: A) no intervention; B) only persons ≥60 years of age; C) persons ≥60 years of age and children; D) adults only; and E) all groups. Thin lines show 1,000 realizations of varying parameters for R_0, γ, and σ (see Methods). Thick lines show the mean simulation, corresponding to $R_0 = 3$, $\gamma = 1/5.015$, $\sigma = 1/5.159$. We assume adults ≥60 years of age will reduce their contact by 95%, children by 85%, and adults by 25%. Dotted lines indicate the beginning of the social distancing intervention at 50 days and ending at 92 days. Age groups include children; persons ≤19 years of age; adults, persons 20–59 years of age; and persons ≥60 years of age.

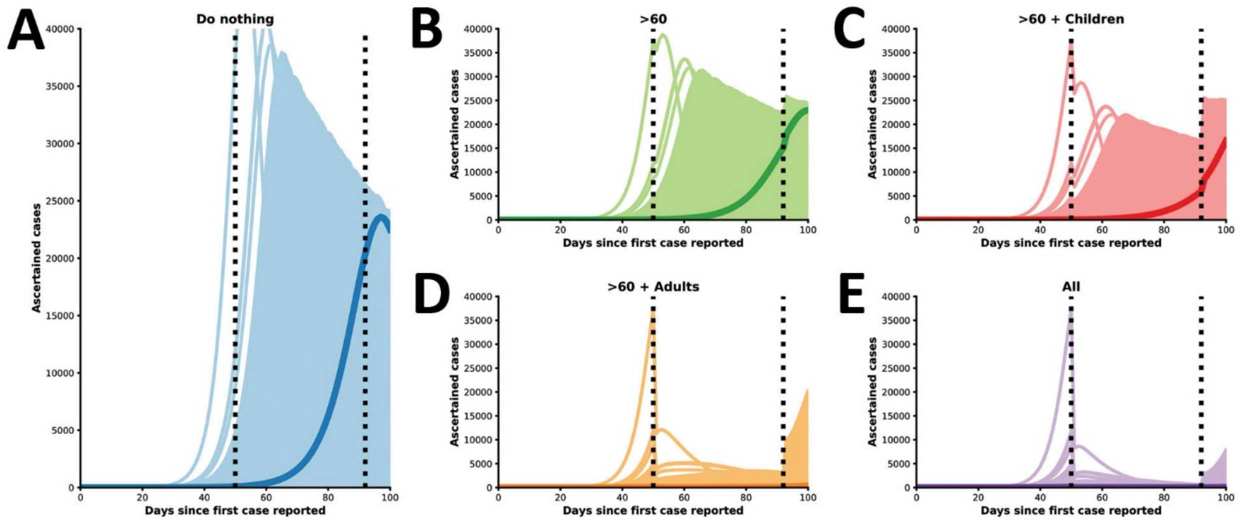

Appendix Figure 2. Number of ascertained coronavirus disease (COVID-19) cases for 5 scenarios of social distancing: A) no intervention; B) only persons ≥60 years of age; C) persons ≥60 years of age and children; D) adults only; and E) all groups. Thin lines show 1,000 realizations of varying parameters for R_0, γ, and σ (see Methods). Thick lines show the mean simulation, corresponding to $R_0 = 3$, $\gamma = 1/5.015$, $\sigma = 1/5.159$. We assume adults ≥60 years of age will reduce their contact by 95%, children by 85%, and adults by 75%. Dotted lines indicate the beginning of the social distancing intervention at 50 days and ending at 92 days. Age groups include children; persons ≤19 years of age; adults, persons 20–59 years of age; and persons ≥60 years of age.

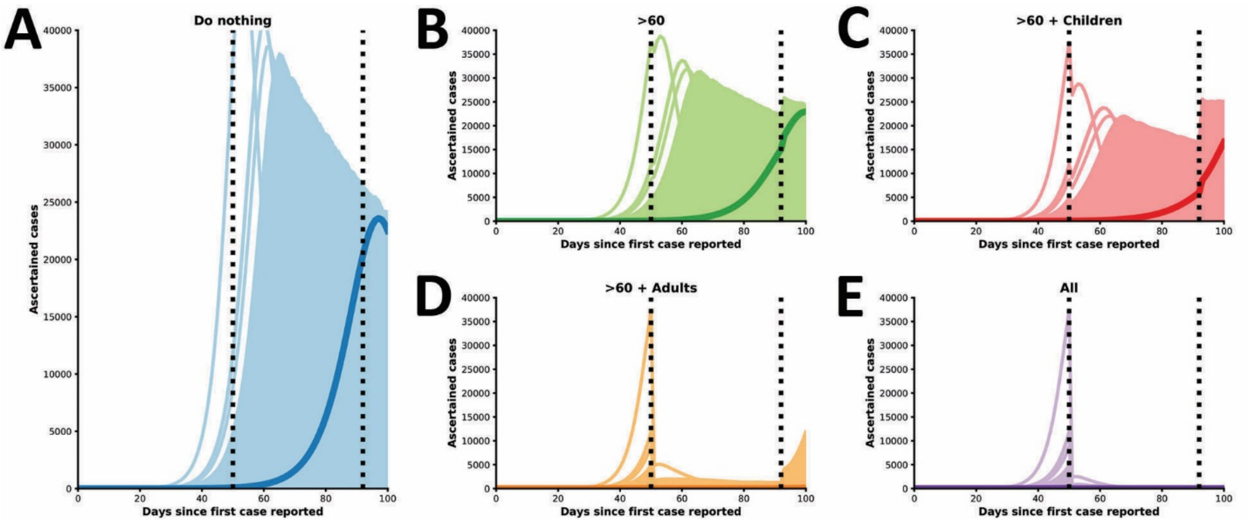

Appendix Figure 3. Number of ascertained coronavirus disease (COVID-19) cases for 5 scenarios of social distancing: A) no intervention; B) only persons ≥60 years of age; C) persons ≥60 years of age and children; D) adults only; and E) all groups. Thin lines show 1,000 realizations of varying parameters for R_0, γ, and σ (see Methods). Thick lines show the mean simulation, corresponding to $R_0 = 3$, $\gamma = 1/5.015$, $\sigma = 1/5.159$. We assume adults ≥60 years of age will reduce their contact by 95%, children by 85%, and adults by 95%. Dotted lines indicate the beginning of the social distancing intervention at 50 days and ending at 92 days. Age groups include children; persons ≤19 years of age; adults, persons 20–59 years of age; and persons ≥60 years of age.

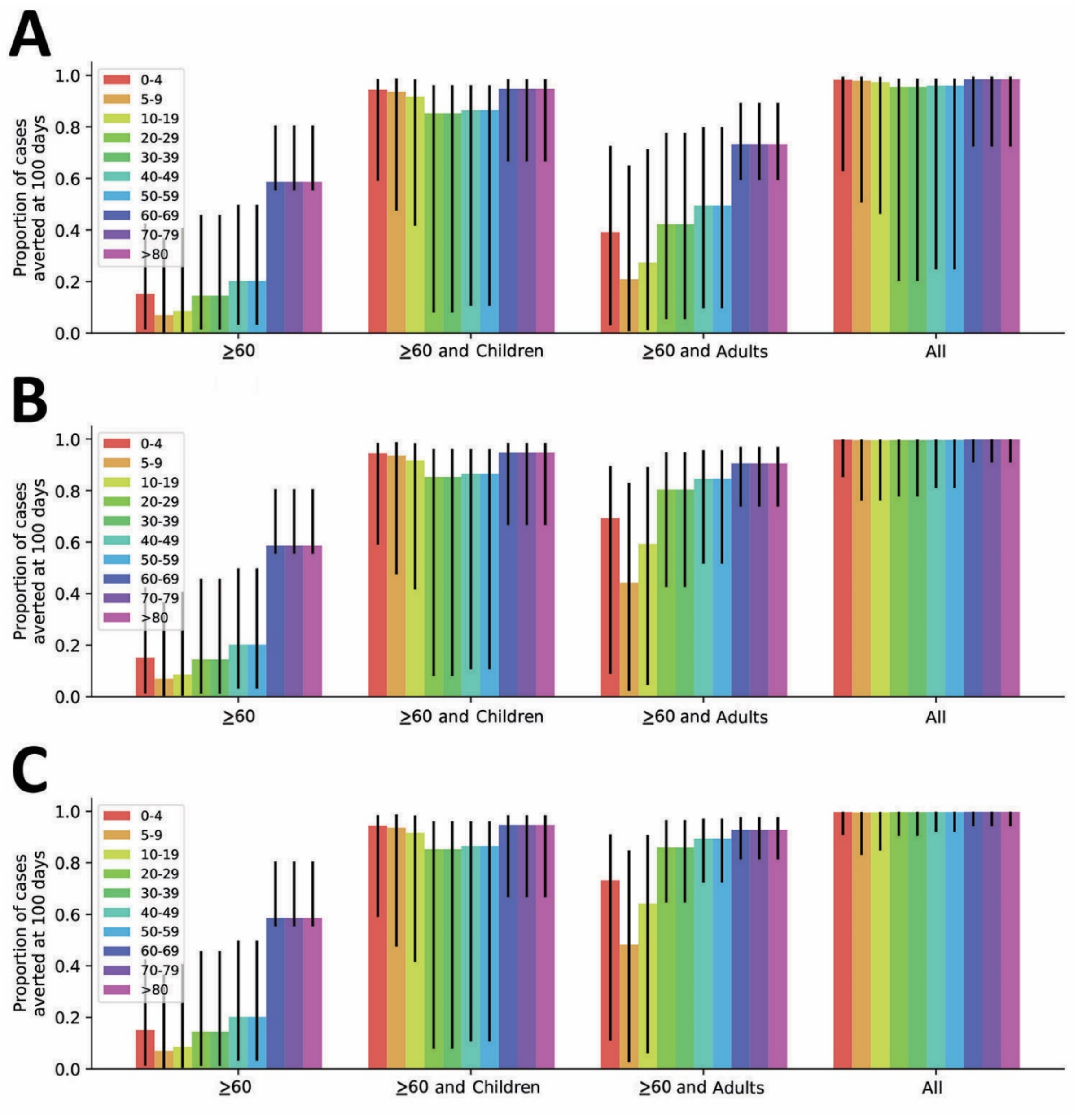

Appendix Figure 4. Proportion of coronavirus disease (COVID-19) cases averted at 100 days under 4 scenarios of persons 20–59 years of age reducing their contact by A) 25%; B) 75%; and C) 95%.

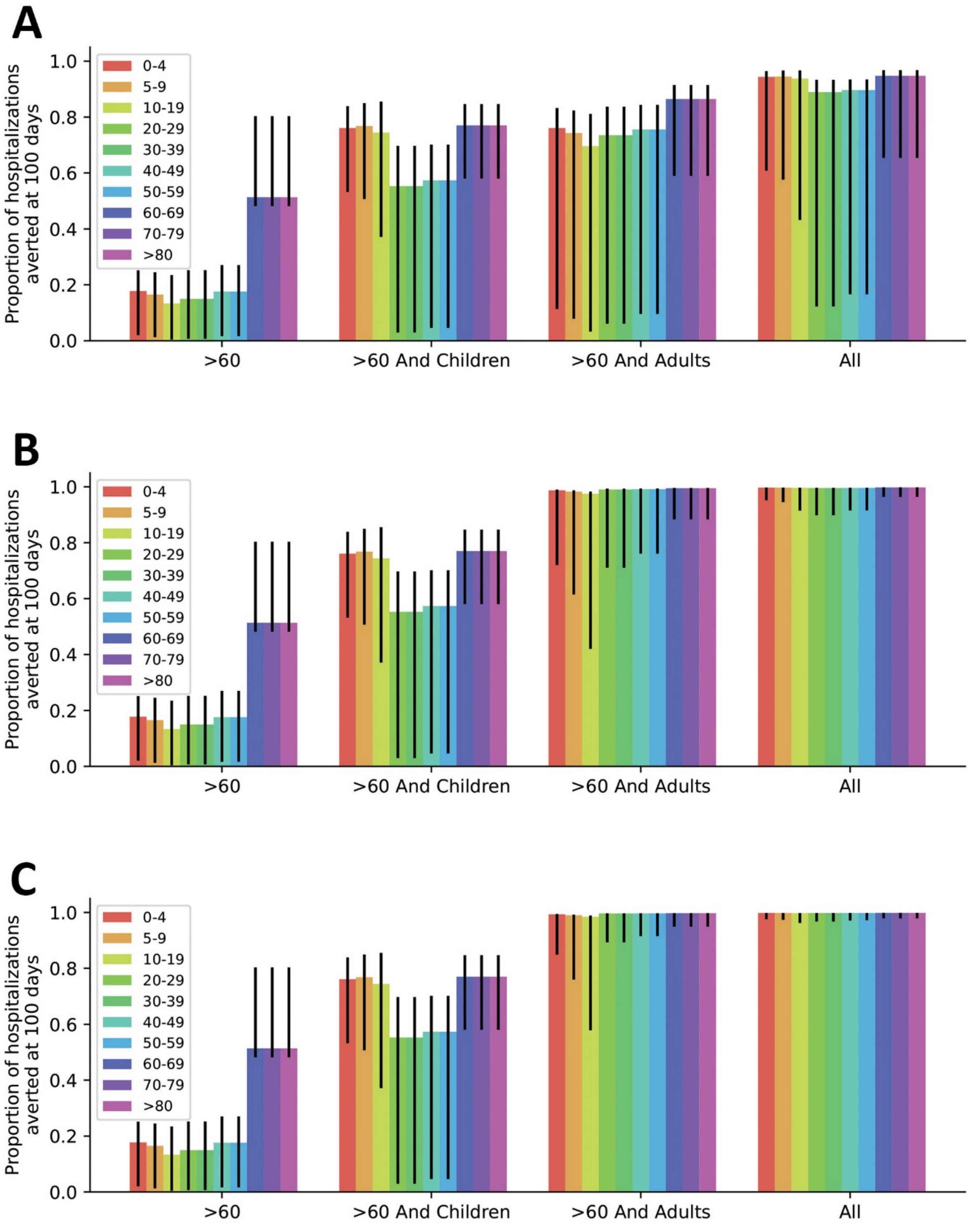

Appendix Figure 5. Proportion of coronavirus disease (COVID-19) hospitalizations averted at 100 days under 4 scenarios of persons 20–59 years of age reducing their contact by A) 25%; B) 75%; and C) 95%.

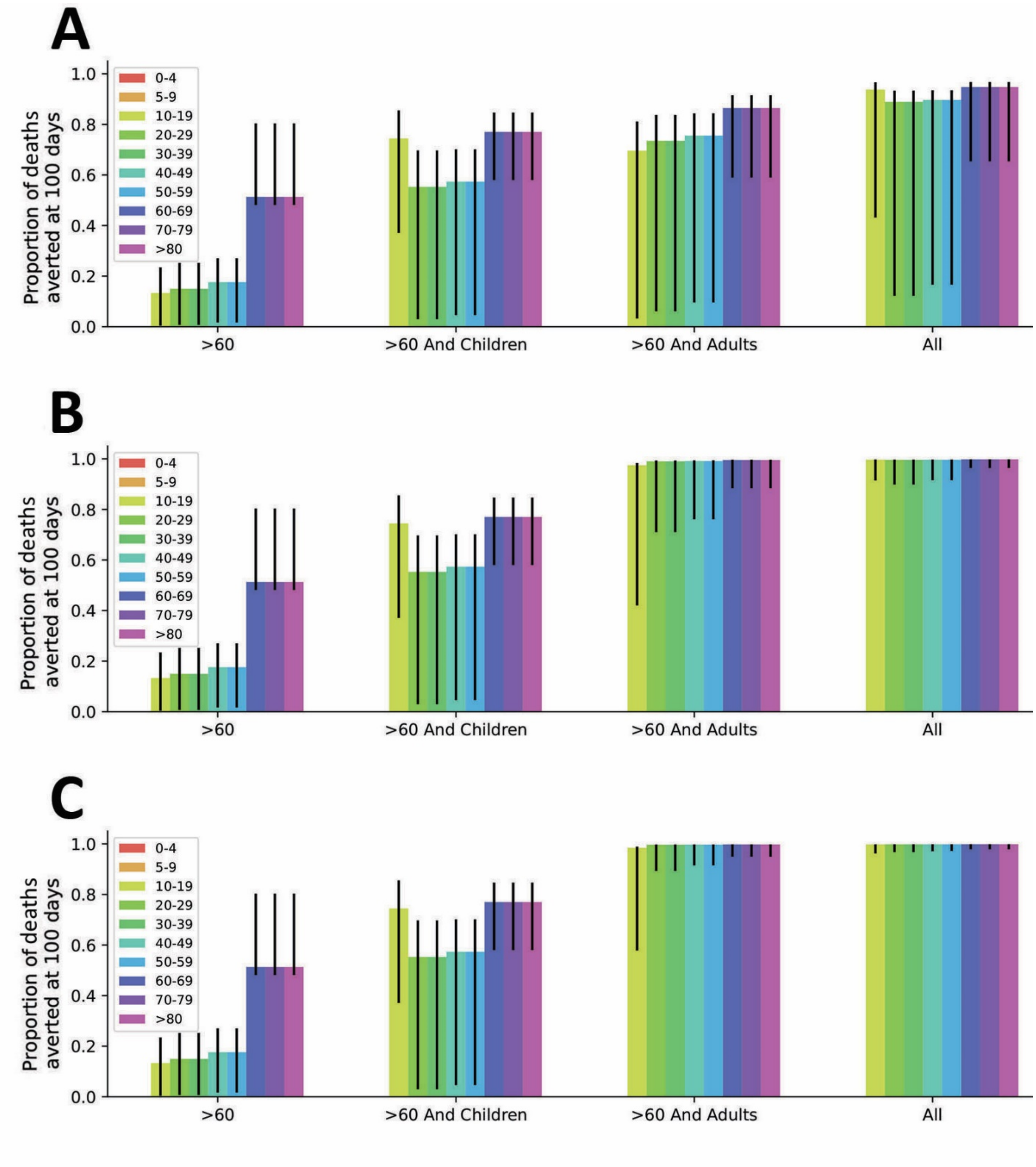

Appendix Figure 6. Proportion of coronavirus disease (COVID-19) deaths averted at 100 days under 4 scenarios of persons 20–59 years of age reducing their contact by A) 25%; B) 75%; and C) 95%.
